# Supplementary material for: Temporal perturbation of ERK dynamics reveals network architecture of FGF2/MAPK signaling
Source: Mol Syst Biol. 2019 Nov 19;15(11):e8947. doi: 10.15252/msb.20198947 (PMC6864398; doi:10.15252/msb.20198947)
Supplement: Supplementary file 1 — Appendix [file MSB-15-e8947-s001.pdf]

Appendix

Temporal perturbation of ERK dynamics reveals network architecture of FGF2-MAPK signaling

Yannick Blum[1]*, Jan Mikelson[2]*, Maciej Dobrzyński[1]*, Hyunryul Ryu[3,4], Marc-Antoine Jacques[1], Noo Li Jeon[3], Mustafa Khammash[2], Olivier Pertz [1**]

Table of content

- Appendix Text
- Appendix Tables
  - Appendix Table S1
  - Appendix Table S2
  - Appendix Table S3
  - Appendix Table S4
  - Appendix Table S5
- Appendix Figures
  - Appendix Figure S1
  - Appendix Figure S2
- Description of Movie EV1
- Appendix dataset availability

Appendix Text

Even though, DTW is the golden standard for shape clustering of time series, we also took additional approaches to confirm that FGF2 evokes a wider mix of ERK signaling states within a cell population. In the 1st approach, we performed PCA analysis on the same pooled responses to EGF/NGF/FGF that we used for clustering (Fig 1E). Figure EV2A shows the two first components that together account for 84% of data variability. The responses to EGF gradually shift only along the 1st principal component (PC) with the increasing GF concentration. The response to EGF 0.25ng/ml is the most dispersed due to the highest cell-cell variability in the decay rate (Materials and Methods, Figure EV1A). NGF responses vary along PC1 and PC2, although the responses to 25 and 250 ng/ml overlap due to saturation. The responses to FGF stimulation overlap the least of all three GFs and vary across both PCs.

In a 2nd approach, we further explored how the dose response challenge of individual GFs might result in diverse single-cell ERK activity temporal patterns. We used a distance-based method to quantify separability between two populations of single-cell time series (Materials and Methods, Fig EV2B). At every time point, we calculate the distance between two distributions of single-cell ERK responses. We then compute the area under curve of that distance over time (Materials and Methods, Fig EV2C), which we use to construct dendrograms to visualize the separation of populations of single-cell responses in response to different GF concentrations (Fig 1H). The responses to EGF and NGF 0.25 ng/ml are the farthest from the remaining responses to EGF and NGF. The two EGF and NGF responses (25 and 250 ng/ml) that overlap in the PCA analysis in panel Figure EV2A, are also deemed close using our distance measure. Finally, FGF2 induces two types of responses located in two distant branches: 1st branch for 0.25 and 2.5 ng/ml that corresponds to a sustained response, and the 2nd branch (25 and 250 ng/ml) that corresponds to an adaptive response followed by a slow recovery towards sustained activity. Together, the PCA and the population distance, confirm a wider range of dynamic ERK signaling states induced by FGF2 in the 4-orders-of-magnitude GF concentration does response, compared to EGF and NGF.

Appendix Table S1: Modeled species, notation and initial value

Species	Notation	Initial Value	Units
RAS	RAS	0.1	μM
Phosphorylated RAS	RAS^*	0	μM
RAF	RAF	0.7	μM
Phosphorylated RAF	RAF^*	0	μM
MEK	MEK	0.68	μM
Phosphorylated MEK	MEK^*	0	μM
ERK	ERK	0.26	μM
Phosphorylated ERK	ERK^*	0	μM
FGF2 receptor	$FGFR$	$fgfr_{init}$	arbitrary units
Active FGF2 receptor	$FGFR^*$	0	arbitrary units
Negative feedback species	NFB	nfb_{init}	arbitrary units
Active negative feedback species	NFB^*	0	arbitrary units
HSPG	H	h_{init}	arbitrary units
HSPG-FGF2	HF	0	arbitrary units
HSPG-FGF2-FGFR	HFR	0	arbitrary units
FGF2 input	$FGFR2$	Input variable	ng/ml

Appendix Table S2: Model equations for basic model

Nbr	Model Equations	Models
1	$\dot{RAS} = -k_{1,2} \cdot HFR \frac{RAS}{K_{1,2} + RAS} + k_{2,1} \frac{RAS^*}{K_{2,1} + RAS^*}$	A1, A2, B1, B2, C1, C2
2	$\dot{RAS}^* = k_{1,2} \cdot HFR \frac{RAS}{K_{1,2} + RAS} - k_{2,1} \frac{RAS^*}{K_{2,1} + RAS^*}$	A1, A2, B1, B2, C1, C2
3	$\dot{RAS} = -\left(k_{1,2} \cdot HFR + r_{1,2} \cdot FGFR^*\right) \frac{RAS}{K_{1,2} + RAS} + k_{2,1} \frac{RAS^*}{K_{2,1} + RAS^*}$	A3, B3, C3, D3
4	$\dot{RAS}^* = \left(k_{1,2} \cdot HFR + r_{1,2} \cdot FGFR^*\right) \frac{RAS}{K_{1,2} + RAS} - k_{2,1} \cdot \frac{RAS^*}{K_{2,1} + RAS^*}$	A3, B3, C3, D3
5	$\dot{RAF} = -k_{3,4} \cdot Ras^* \frac{Raf}{K_{3,4} + Raf} + k_{4,3} \frac{Raf^*}{K_{4,3} + Raf^*}$	A1, A2, A3
6	$\dot{RAF}^* = k_{3,4} \cdot RAS^* \frac{RAF}{K_{3,4} + Raf} - k_{4,3} \frac{RAF^*}{K_{4,3} + RAF^*}$	A1, A2, A3
7	$\dot{RAF} = -k_{3,4} Ras^* \frac{Raf}{K_{3,4} + Raf} \frac{K_{NFB}^{h_{nfb}}}{K_{NFB}^{h_{nfb}} + NFB^{*h_{nfb}}} + k_{4,3} \frac{Raf^*}{K_{4,3} + Raf^*}$	B1, B2, B3, C1, C2, C3, D1, D2, D3
8	$\dot{RAF}^* = k_{3,4} \cdot RAS^* \frac{RAF}{K_{3,4} + Raf} \frac{K_{NFB}^{h_{nfb}}}{K_{NFB}^{h_{nfb}} + NFB^{*h_{nfb}}} - k_{4,3} \frac{RAF^*}{K_{4,3} + RAF^*}$	B1, B2, B3, C1, C2, C3, D1, D2, D3
9	$\dot{MEK} = -k_{5,6} \cdot RAF^* \frac{MEK}{K_{5,6} + MEK} + k_{6,5} \frac{MEK^*}{K_{6,5} + MEK^*}$	All models
10	$\dot{MEK}^* = k_{5,6} \cdot RAF^* \frac{MEK}{K_{5,6} + MEK} - k_{6,5} \frac{MEK^*}{K_{6,5} + MEK^*}$	All models
11	$\dot{ERK} = -k_{7,8} \cdot MEK^* \frac{ERK}{K_{7,8} + ERK} + k_{8,7} \frac{ERK^*}{K_{8,7} + ERK^*}$	All models
12	$\dot{ERK}^* = k_{7,8} \cdot MEK^* \frac{ERK}{K_{7,8} + ERK} - k_{8,7} \frac{ERK^*}{K_{8,7} + ERK^*}$	All models

Appendix Table S3: Model equations for receptor models.

Nbr	Model Equations	Models
13	$\dot{FGFR} = -r_{5,7} \cdot HF \cdot FGFR + r_{7,5} \cdot HFR$	A1, B1, C1, D1
14	$\dot{FGFR} = -r_{3,4} \cdot FGF2 \cdot FGFR + r_{4,3} \cdot FGFR^{*} - r_{5,7} \cdot HF \cdot FGFR + r_{7,5} \cdot HFR$	A2, A3, B2, B3, C2, C3, D2, D3
15	$\dot{FGFR}^{*} = r_{3,4} \cdot FGF2 \cdot FGFR - r_{4,3} \cdot FGFR^{*}$	A2, A3, B2, B3, C2, C3, D2, D3
16	$\dot{H} = -r_{5,6} \cdot H \cdot FGF2 + r_{6,5} \cdot HF$	All models
17	$\dot{HF} = \left(r_{5,6} \cdot H \cdot FGF2 + r_{7,5} \cdot HFR\right) - \left(r_{6,5} \cdot HF + r_{5,7} \cdot HF \cdot FGFR\right)$	All models
18	$\dot{HFR} = r_{5,7} \cdot HF \cdot FGFR - r_{7,5} \cdot HFR$	All models

Appendix Table S4: Model equations for feedback models.

Nbr	Model Equations	Models
19	$\dot{NFB} = -f_{1,2} \cdot ERK^{*} \frac{NFB}{F_{1,2} + NFB} + f_{2,1} \frac{NFB^{*}}{F_{2,1} + NFB^{*}}$	B1, B2, B3
20	$\dot{NFB}^{*} = f_{1,2} \cdot ERK^{*} \frac{NFB}{F_{1,2} + NFB} - f_{2,1} \frac{NFB^{*}}{F_{2,1} + NFB^{*}}$	B1, B2, B3
21	$\dot{NFB} = -f_{1,2} \frac{NFB}{F_{1,2} + NFB} \frac{HFR^{h_{hfr}}}{F_{hfr}^{h_{hfr}} + HFR_{hfr}^{h}} + f_{2,1} \frac{NFB^{*}}{F_{2,1} + NFB^{*}}$	C1, C2
22	$\dot{NFB}^{*} = f_{1,2} \frac{NFB}{F_{1,2} + NFB} \frac{HFR^{h_{hfr}}}{F_{hfr}^{h_{hfr}} + HFR_{hfr}^{h}} - f_{2,1} \frac{NFB^{*}}{F_{2,1} + NFB^{*}}$	C1, C2
23	$\dot{NFB} = -f_{1,2} \frac{NFB}{F_{1,2} + NFB} \left(f_h \frac{HFR^{h_{hfr}}}{F_{hfr}^{h_{hfr}} + HFR^{h_{hfr}}} + (1 - f_h) \frac{FGFR^{*h_{fgfr}}}{F_{fgfr}^{h_{fgfr}} + FGFR^{*h_{fgfr}}} \right)$<br>$+f_{2,1} \frac{Nfb^{*}}{F_{2,1} + NFB^{*}}$	C3
24	$\dot{NFB}^{*} = f_{1,2} \frac{NFB}{F_{1,2} + NFB} \left(f_h \frac{HFR^{h_{hfr}}}{F_{hfr}^{h_{hfr}} + HFR^{h_{hfr}}} + (1 - f_h) \frac{FGFR^{*h_{fgfr}}}{F_{fgfr}^{h_{fgfr}} + FGFR^{*h_{fgfr}}} \right)$<br>$-f_{2,1} \frac{Nfb^{*}}{F_{2,1} + NFB^{*}}$	C3
25	$\dot{NFB} = -f_{1,2} \frac{NFB}{F_{1,2} + NFB} \frac{HFR^{h_{hfr}}}{F_{hfr}^{h_{hfr}} + HFR_{hfr}^{h}} + \frac{f_{2,1} * NFB^{*}}{F_{2,1} + NFB^{*}}$	D1, D2
26	$\dot{NFB}^{*} = f_{1,2} \cdot ERK^{*} \cdot \frac{NFB}{F_{1,2} + NFB} \frac{HFR^{h_{hfr}}}{F_{hfr}^{h_{hfr}} + HFR_{hfr}^{h}} - \frac{f_{2,1} * NFB^{*}}{F_{2,1} + NFB^{*}}$	D1, D2
27	$\dot{NFB} = -f_{1,2} \cdot ERK^{*} \cdot \frac{NFB}{F_{1,2} + NFB}$<br>$\left(f_h \frac{HFR^{h_{hfr}}}{F_{hfr}^{h_{hfr}} + HFR^{h_{hfr}}} + (1 - f_h) \frac{FGFR^{*h_{fgfr}}}{F_{fgfr}^{h_{fgfr}} + FGFR^{*h_{fgfr}}} \right) + f_{2,1} \frac{NFB^{*}}{F_{2,1} + NFB^{*}}$	D3
28	$\dot{NFB}^{*} = f_{1,2} \cdot ERK^{*} \cdot \frac{NFB}{F_{1,2} + NFB} \left(f_h \frac{HFR^{h_{hfr}}}{F_{hfr}^{h_{hfr}} + HFR^{h_{hfr}}} + (1 - f_h) \frac{FGFR^{*h_{fgfr}}}{F_{fgfr}^{h_{fgfr}} + FGFR^{*h_{fgfr}}} \right)$<br>$-f_{2,1} \frac{NFB^{*}}{F_{2,1} + NFB^{*}}$	D3

Appendix Table S5: Description of model parameters.

Nbr	Parameter Name	Description	Units
1	$k_{1,2}$	HFR dependent RAS phosphorylation rate	s^{-1}
2	$K_{1,2}$	Michaelis constant RAS phosphorylation	μM
3	$k_{2,1}$	RAS dephosphorylation rate	s^{-1}
4	$K_{2,1}$	Michaelis constant RAS dephosphorylation	μM
5	$r_{1,2}$	FGFR dependent RAS phosphorylation rate	s^{-1}
6	$k_{3,4}$	RAF phosphorylation rate	s^{-1}
7	$K_{3,4}$	Michaelis constant RAF phosphorylation	μM
8	$k_{4,3}$	RAF dephosphorylation rate	s^{-1}
9	$K_{4,3}$	Michaelis constant RAF dephosphorylation	μM
10	h_{nfb}	Hill exponent for NFB effect on RAF phosphorylation	-
11	K_{nfb}	Hill constant for NFB effect on RAF phosphorylation	-
12	$k_{5,6}$	RAF dependent MEK phosphorylation rate	s^{-1}
13	$K_{5,6}$	Michaelis constant MEK phosphorylation	μM
14	$k_{6,5}$	MEK dephosphorylation rate	s^{-1}
15	$K_{6,5}$	Michaelis constant MEK dephosphorylation	μM
16	$k_{7,8}$	MEK dependent ERK phosphorylation rate	s^{-1}
17	$K_{7,8}$	Michaelis constant ERK phosphorylation	μM
18	$k_{8,7}$	ERK dephosphorylation rate	s^{-1}
19	$K_{8,7}$	Michaelis constant ERK dephosphorylation	μM
20	$r_{5,7}$	FGFR binding from HSPG-FGF2	s^{-1}
21	$r_{7,5}$	FGFR unbinding from HSPG-FGF2	s^{-1}
22	$r_{3,4}$	FGF2 dependent FGFR activation rate	s^{-1}
23	$r_{4,3}$	FGFR deactivation rate	s^{-1}
24	$r_{5,6}$	Binding rate of HSPG to FGF2	s^{-1}
25	$r_{6,5}$	Unbinding rate of HSPG to FGF2	s^{-1}
26	$f_{1,2}$	ERK dependent NFB activation rate	s^{-1}
27	$F_{1,2}$	Michaelis constant NFB activation	-
28	$f_{2,1}$	NFB deactivation rate	s^{-1}
29	$F_{2,1}$	Michaelis constant NFB deactivation	-

30	h_{hfr}	Hill exponent for HFR dependent NFB activation	-
31	F_{hfr}	Hill constant for HFR dependent NFB activation	-
32	f_h	Ratio of HSPG-FGF2 contribution to RAS activation	-
33	h_{fgfr}	Hill exponent for FGFR dependent NFB activation	-
34	F_{fgfr}	Hill constant for FGFR dependent NFB activation	-
35	$fgfr_{init}$	Initial state for FGFR	arbitrary units
36	nfb_{init}	Initial state for NFB	arbitrary units
37	h_{init}	Initial state for HSPG	arbitrary units

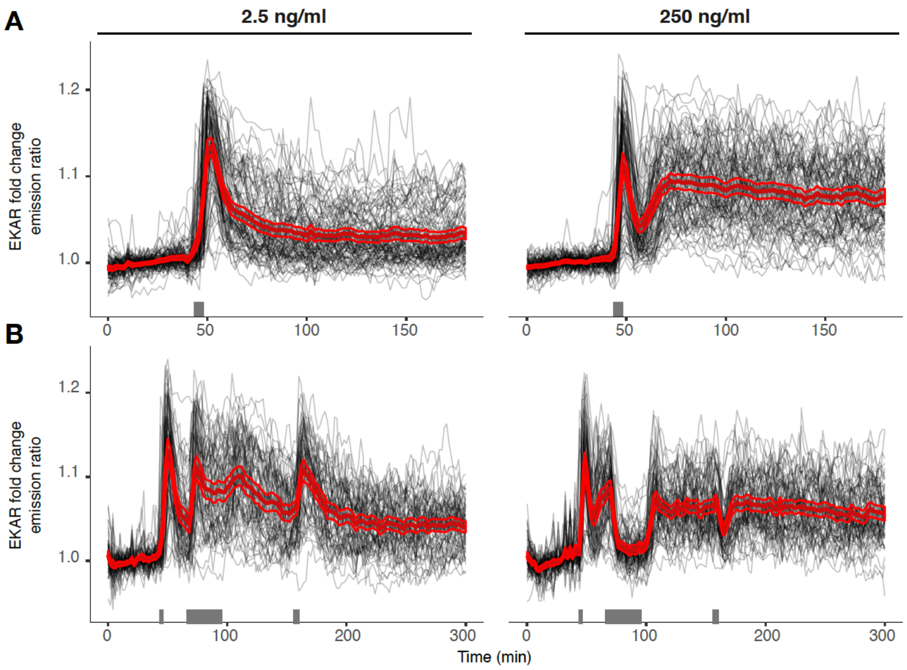

Appendix Figure S1. Dynamic FGF2 stimulation datasets for candidate model prediction.

Single-cell trajectories in response to 2.5 and 250 ng/m FGF2 5' single pulse (A), and a mixed pulse stimulation scheme consisting of 3' pulse - 20' pause - 30' pulse - 50' pause - 5' pulse (B). Solid red line – population mean, thin red lines – 95% CI for the mean. Trajectories were normalized to their own means at t=[0, 40] min.

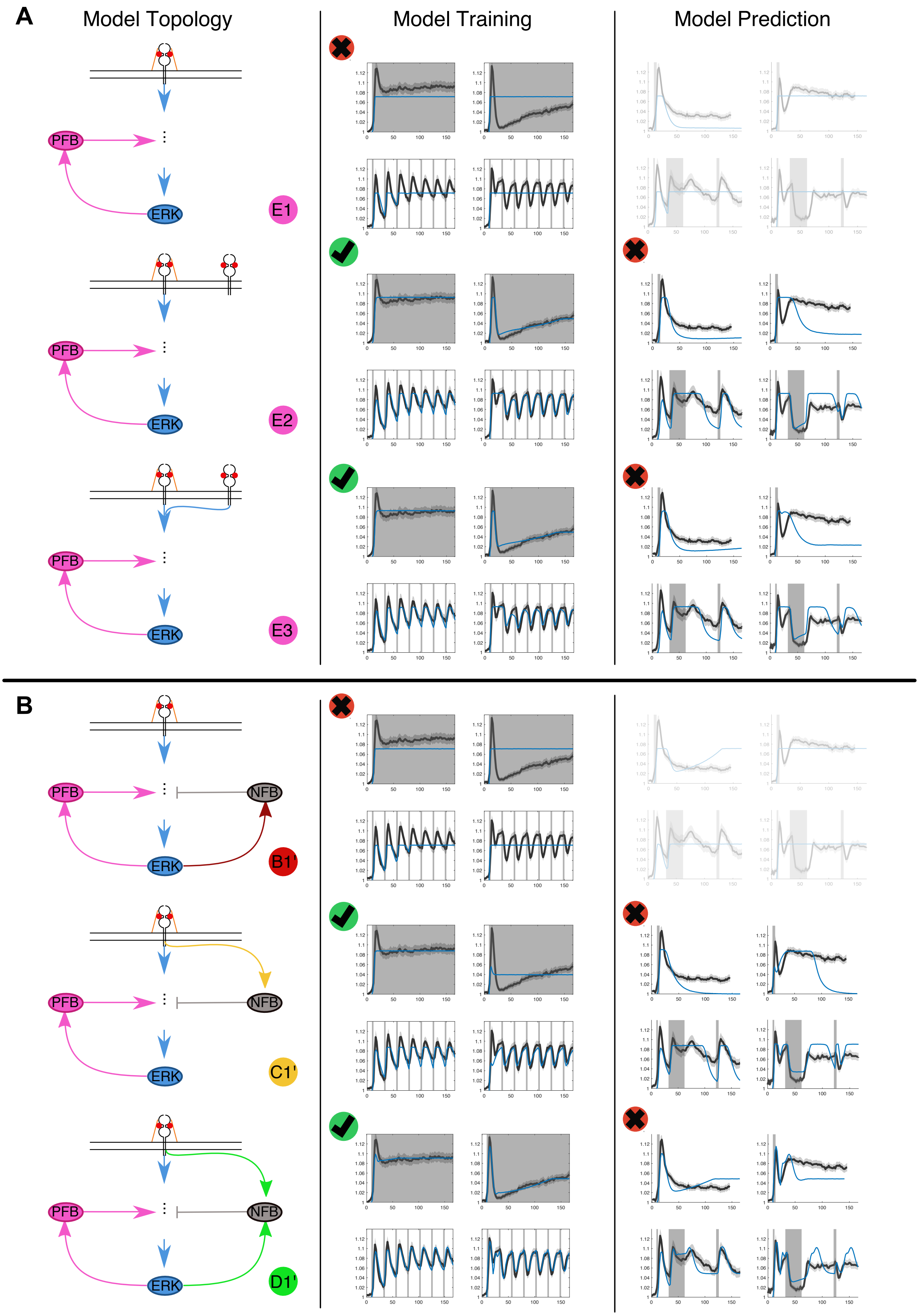

Appendix Figure S2. Training and prediction of internal model topologies with positive feedback loop.

Alternative model topologies with positive feedback loops. (A) Models E1, E2 and E3 have the 3 different extracellular receptor models with a simple intracellular topology with a positive feedback only. (B) Models B1’, C1’ and D1’ are variations of models B1, C1 and D1 shown in Fig 6C with added positive feedback. (Middle column) Each model was trained on ERK activity population averages of the following experimental datasets: 2.5ng/ml sustained FGF2 stimulation (top left), 250ng/ml sustained FGF2 stimulation (top right), 2.5ng/ml pulse stimulation (bottom left) and 250ng/ml pulse stimulation (bottom right). (Right column) Each model was simulated and compared with the ERK activity population averages of the following experimental datasets: 2.5 ng/ml FGF2 single 5’ pulse (top left), 250 ng/ml FGF2 single 5’ pulse (top right), 2.5 ng/ml FGF2 mixed pulse (bottom left), 250 ng/ml FGF2 mixed pulse (bottom right). Experimental ERK activity population averages: black lines – 95% CI (grey shaded area). GF stimulation: light-grey vertical areas. Best prediction for of each model (maximum likelihood of training): blue lines. Prediction for models E1 (A) and B1’ (B) are shown in faded colors since they already failed at the model training stage. Green check/red cross symbols indicate satisfying/unsatisfying fits to the training or validation datasets as evaluated by visual inspection.

Movie EV1. ERK activity dynamics in response to 2.5 and 250 ng/ml 3’ pulse/20’ pause multi-pulse FGF2 stimulation scheme.

EKAR2G ratio movie showing responses of two selected cells stimulated with 250ng/ml FGF2 (top) and 2.5ng/ml FGF2 (bottom) to a 3’-20’ multi-pulse regime. FGF2 pulse is visualized using the red dextran appearing between the cells. Scale bar = 10 μm.

Appendix dataset availability

The data archive available at
https://data.mendeley.com/datasets/ccnxn84w8z/1
contains:

1. CSV files with single-cell timeseries for all GF stimulation conditions.
2. CSV files with single-cell differentiation data

3. CellProfiler 2.2.1 pipelines for analysis of time-lapse and differentiation experiments.
