# Supplementary material for: Temporal perturbation of ERK dynamics reveals network architecture of FGF2/MAPK signaling
Source: Mol Syst Biol. 2019 Nov 19;15(11):e8947. doi: 10.15252/msb.20198947 (PMC6864398; doi:10.15252/msb.20198947)
Supplement: Supplementary file 2 — Expanded View Figures PDF [file MSB-15-e8947-s002.pdf]

Expanded View Figures

Figure EV1. Raw single-cell ERK activity trajectories of the EGF, NGF, and FGF dose response.

A Single-cell ERK activity measured at 10 min before and after sustained GF stimulation (T = 30′ and 50′). Single-cell trajectories were normalized to their own means before GF stimulation, t = [0, 40]. Lower and upper hinge correspond to 25th and 75th percentiles. Lower and upper whiskers correspond to 1.5*IQR from the hinge. Replicates: 1.

B ERK activity dynamics in response to stimulation with a dose–response challenge using 0.25, 2.5, 25, and 250 ng/ml EGF, NGF, and FGF2. Single-cell time series were normalized to their own means before GF stimulation, t = [36, 40] min. Red curve indicates the population mean; black horizontal bar indicates GF stimulation. N = [48, 120] cells per GF concentration. ERK dynamics measured at 2′ intervals.

C Single-cell ERK trajectories within 6 clusters identified in Fig 1F. We used hierarchical clustering with dynamic time warping and Ward's linkage method for building the dendrogram, which was then cut to distinguish 6 clusters.

D Hierarchical clustering of individual GF dose–response challenges from (B) with the same approach as (B) but for a longer interval (t = [36, 200] min). The left column indicates cluster means and 95% CI for the mean, and the right column is the distribution of single-cell trajectories across 4 clusters, representative of 3 replicates.

 © 2019 The Authors

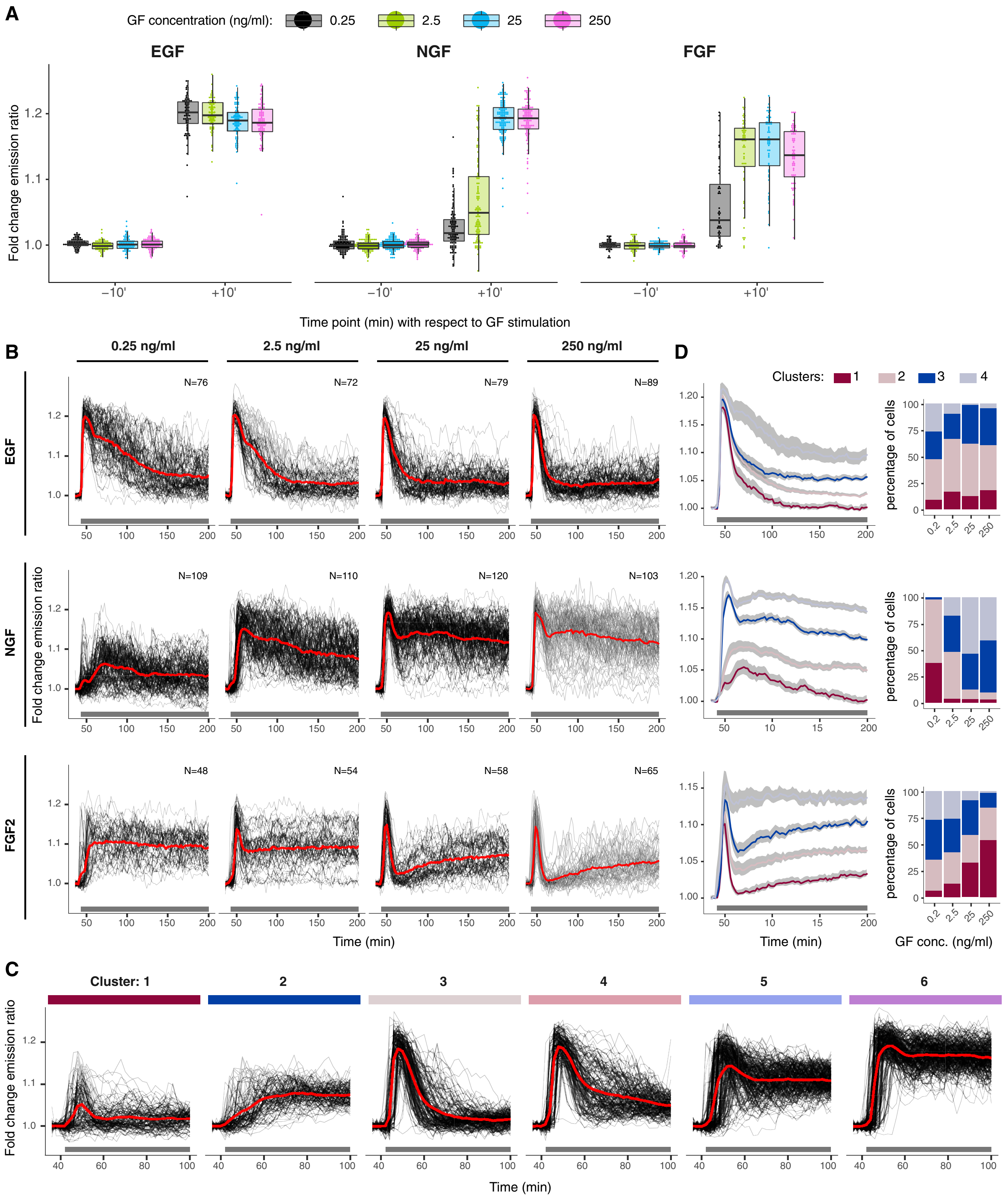
A
GF concentration (ng/ml): 0.25 2.5 25 250
EGF
NGF
FGF
Fold change emission ratio
Time point (min) with respect to GF stimulation
B
0.25 ng/ml
2.5 ng/ml
25 ng/ml
250 ng/ml
EGF
NGF
FGF2
Fold change emission ratio
Time (min)
D
Clusters: 1 2 3 4
percentage of cells
Time (min)
GF conc. (ng/ml)
C
Cluster: 1
2
3
4
5
6
Fold change emission ratio
Time (min)

Figure EV1.

Figure EV2. Separability of ERK states in the EGF, NGF, and FGF2 dose–response challenge.

A Principal component analysis (PCA) of a pooled dataset from Fig 1E. The first two components account for 85% of the overall variability. Here, we use the same data trimmed to t = [36,100] min as in clustering in Fig 1E. Note that in addition to GF responses, responses of untreated cells are also shown, although they were not used in the decomposition (red points).

B Schematic of distance calculation between two populations of single-cell time series. Two synthetic populations of noisy time series data consist of 100 trajectories each, with each trajectory spanning t = [0, 5] T with 0.1 T interval. Step 1: At every measured time point, calculate Jeffries–Matusita distance (d_{JM}) between two distributions of a measured quantity. Step 2: Calculate area under the curve (AUC) of d_{JM} and express it as fraction of the maximum AUC of d_{JM}, which is $2dxN$, where dx is the interval length and N is the number of measured time points. The normalized AUC of d_{JM} is used to construct dendrogram in Fig 1H.

C d_{JM} over time for each GF concentration pair (0.25, 2.5, 25, 250 ng/ml) for EGF, NGF, and FGF2 stimulation. Here, we use the same data trimmed to t = [36,200] min as in Figure EV1B.

 © 2019 The Authors

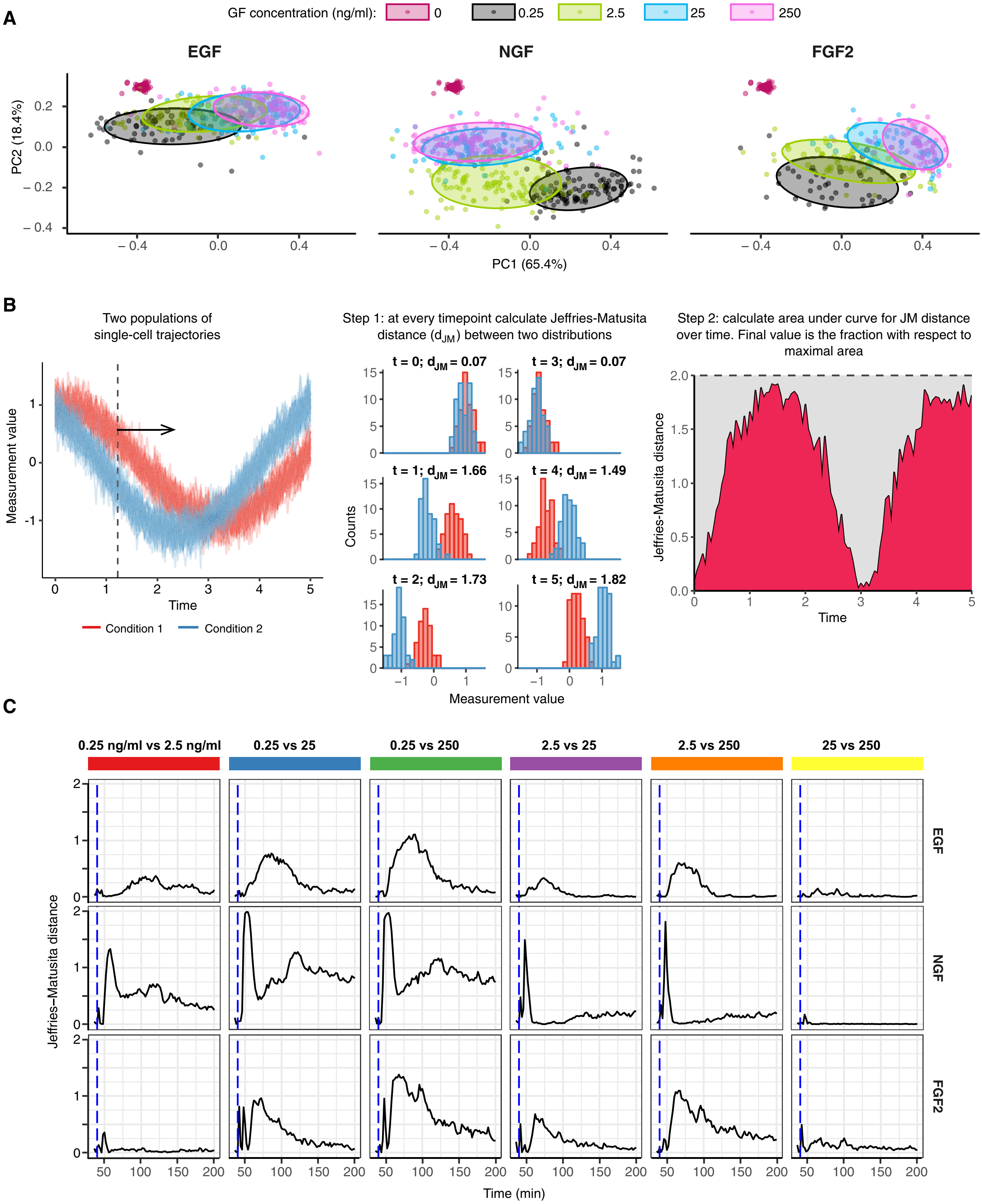
A
GF concentration (ng/ml): 0 0.25 2.5 25 250
EGF
NGF
FGF2
PC2 (18.4%)
PC1 (65.4%)
B
Two populations of single-cell trajectories
Measurement value
Time
Condition 1
Condition 2
Step 1: at every timepoint calculate Jeffries-Matusita distance (dJM) between two distributions
t = 0; dJM = 0.07
t = 3; dJM = 0.07
t = 1; dJM = 1.66
t = 4; dJM = 1.49
t = 2; dJM = 1.73
t = 5; dJM = 1.82
Counts
Measurement value
Step 2: calculate area under curve for JM distance over time. Final value is the fraction with respect to maximal area
Jeffries-Matusita distance
Time
C
0.25 ng/ml vs 2.5 ng/ml
0.25 vs 25
0.25 vs 250
2.5 vs 25
2.5 vs 250
25 vs 250
EGF
NGF
FGF2
Jeffries–Matusita distance
Time (min)

Figure EV2.

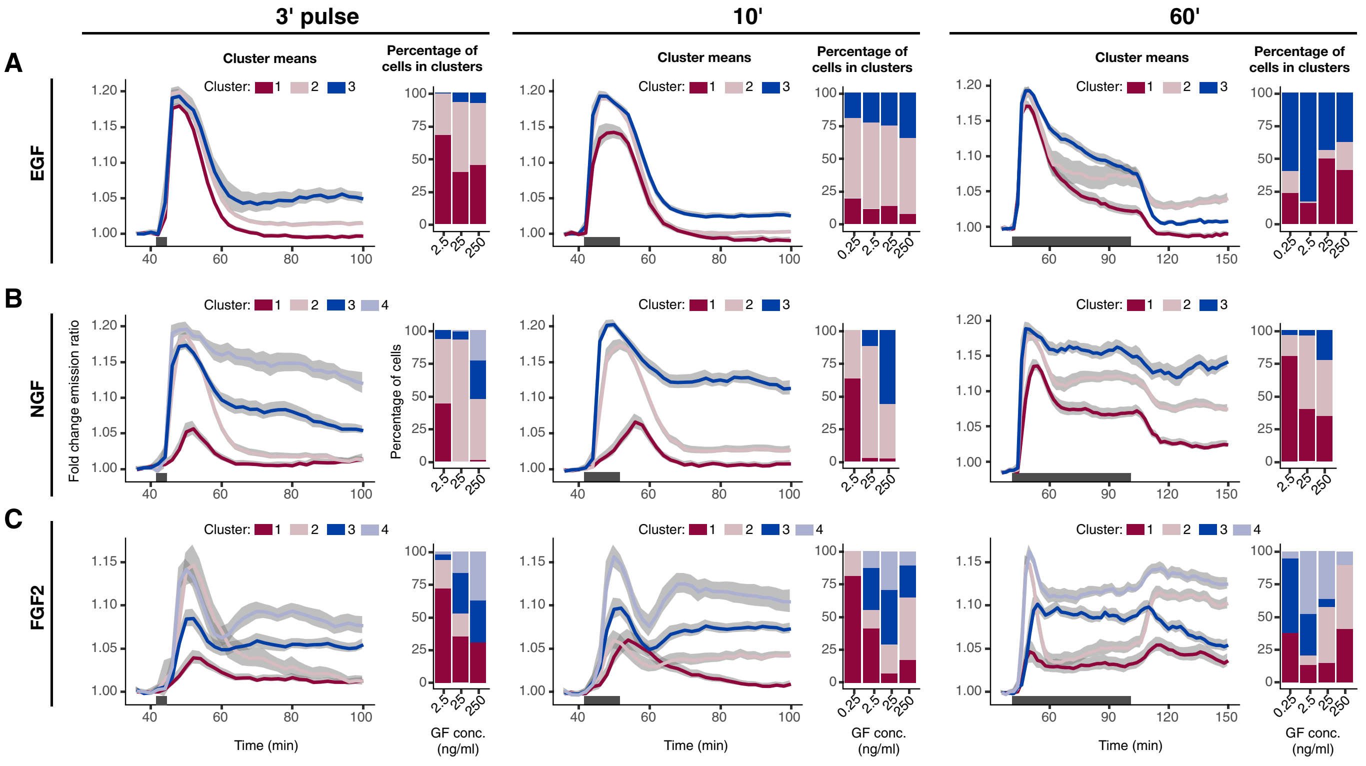

Figure EV3. Clustering of dynamic ERK activity signaling states in response to single-pulse GF stimulation regimes.

A–C Hierarchical clustering of dynamic ERK responses to a single 3′, 10′, and 60′ pulse of EGF (A), NGF (B), and FGF2 (C). We used dynamic time warping and Ward's linkage method for building the dendrogram, which was then cut to highlight 3 or 4 main branches with major dynamic patterns. GF dose responses in each panel were clustered independently; thus, cluster averages that share the same color across panels are unrelated.

 © 2019 The Authors

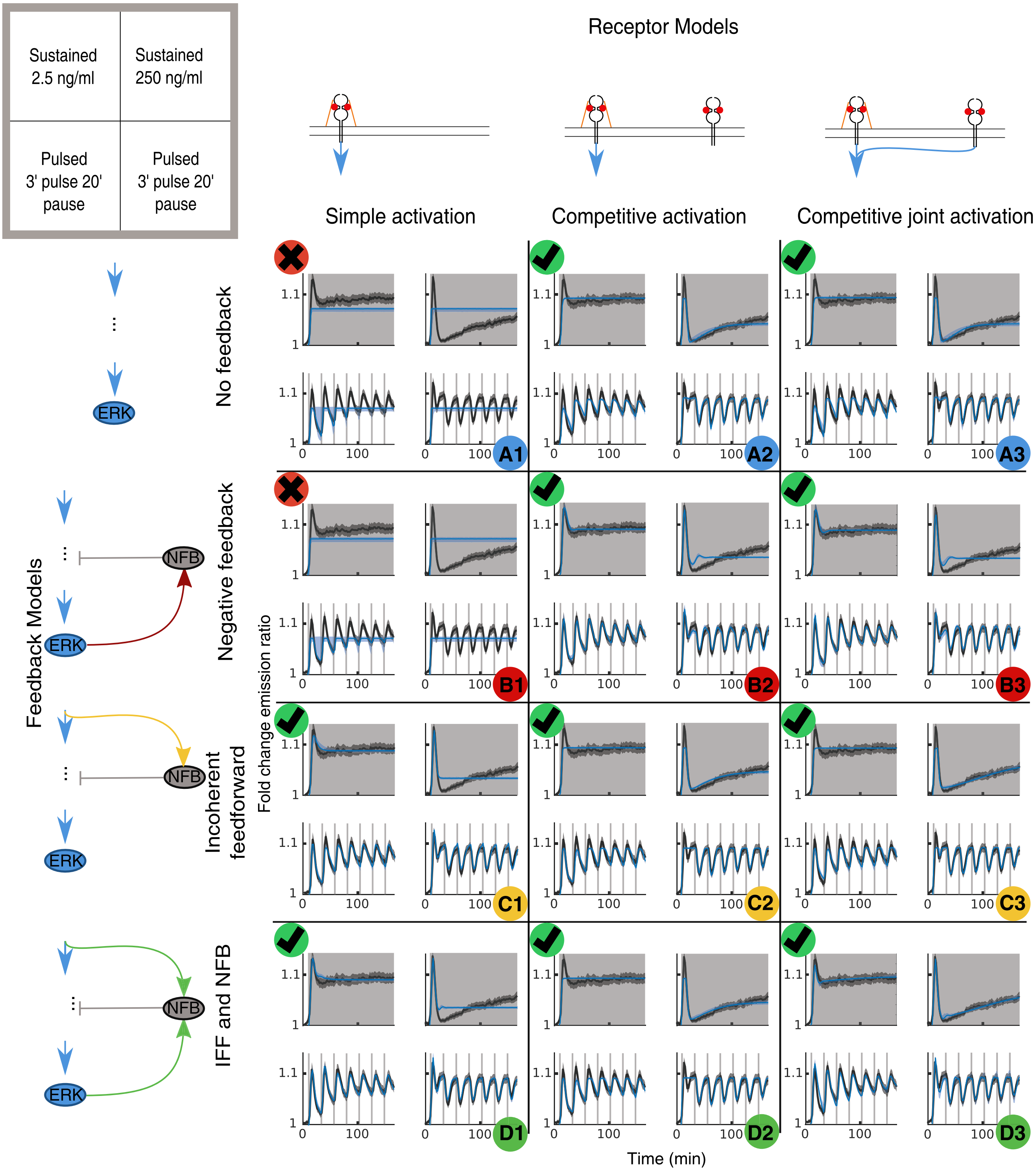

Figure EV4. Candidate model training.

Model training for all proposed candidate models. Each model was trained on ERK activity population averages of the following experimental datasets (as shown in the upper left key): 2.5 ng/ml sustained FGF2 stimulation (top left), 250 ng/ml sustained FGF2 stimulation (top right), 2.5 ng/ml pulse stimulation (bottom left), and 250 ng/ml pulse stimulation (bottom right), representative of 2 replicates for pulse stimulation and 3 replicates for sustained stimulation. Experimental ERK activity population averages: black lines—95% CI (gray shaded area). GF stimulation: light-gray vertical areas. Best fit for of each model (maximum likelihood of training): blue lines. Envelope of the simulations based on parameters sampled from the posterior distribution: light-blue areas. Green check/red cross symbols indicate satisfying/unsatisfying fits to training datasets as evaluated by visual inspection.

© 2019 The Authors

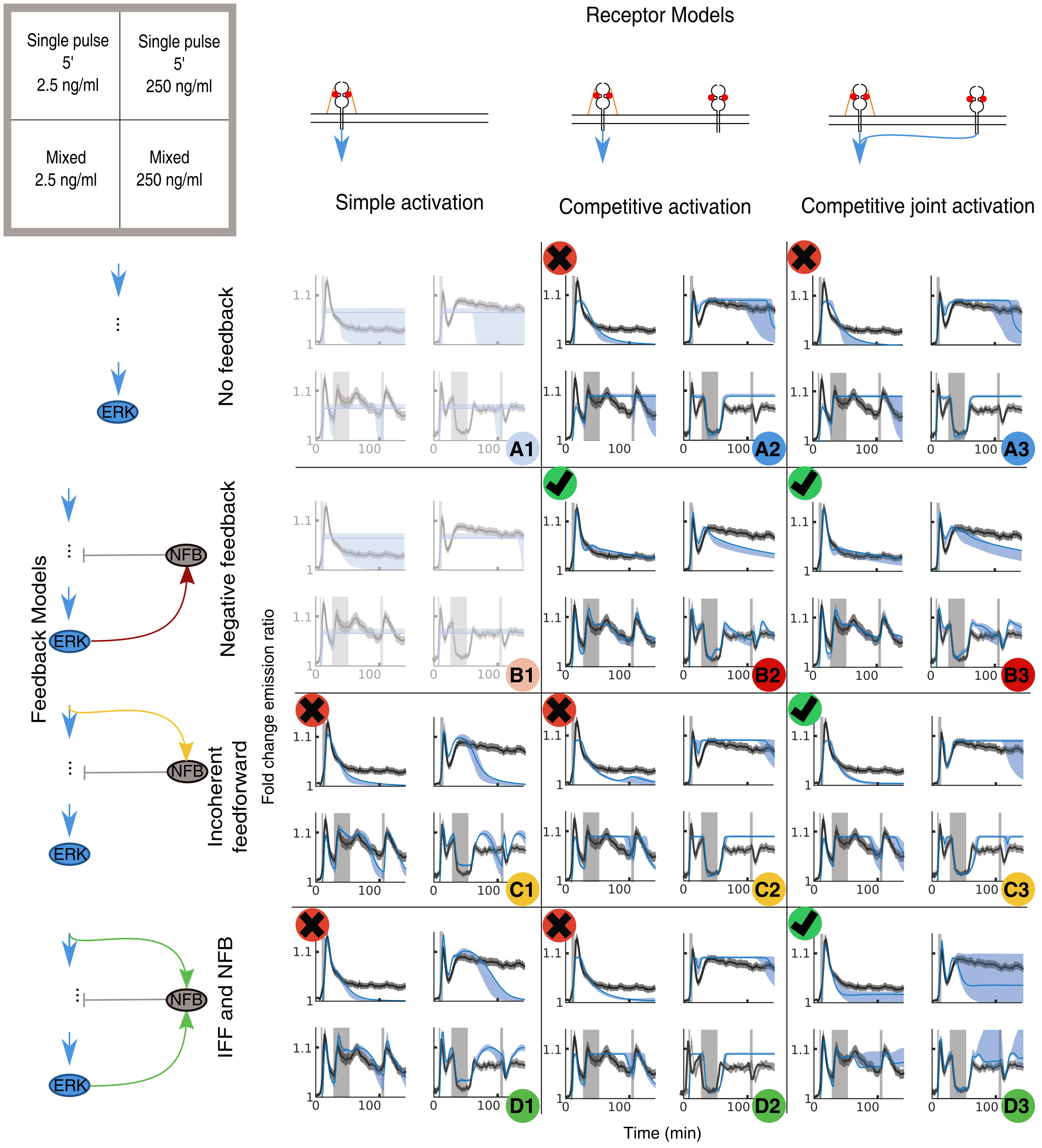

Figure EV5. Candidate model prediction.

Each model was simulated and compared with the ERK activity population averages of the following experimental datasets (as shown in the upper left key): 2.5 ng/ml FGF2 single 5′ pulse (top left), 250 ng/ml FGF2 single 5′ pulse (top right), 2.5 ng/ml FGF2 mixed pulse (bottom left), and 250 ng/ml FGF2 mixed pulse (bottom right). Experimental ERK activity population averages: black lines—95% CI (gray shaded area). GF stimulation: light-gray vertical areas, replicates 1. Best prediction for of each model (maximum likelihood of training): blue lines. Envelope of the simulations based on parameters sampled from the posterior distribution: light-blue areas. Models shown in faded colors indicate failure of parameter inference during training. Green check/red cross symbols indicate satisfying/unsatisfying fits to validation datasets as evaluated by visual inspection.

© 2019 The Authors
