# Supplementary material for: Temporal perturbation of ERK dynamics reveals network architecture of FGF2/MAPK signaling
Source: Mol Syst Biol. 2019 Nov 19;15(11):e8947. doi: 10.15252/msb.20198947 (PMC6864398; doi:10.15252/msb.20198947)
Supplement: Supplementary file 3 — Movie EV1 [file MSB-15-e8947-s003.zip › Movie_EV1.docx]

**Movie EV1. ERK activity dynamics in response to 2.5 and 250 ng/ml 3’ pulse/20’ pause multi-pulse FGF2 stimulation scheme.**

EKAR2G ratio movie showing responses of two selected cells stimulated with 250ng/ml FGF2 (top) and 2.5ng/ml FGF2 (bottom) to a 3’-20’ multi-pulse regime. FGF2 pulse is visualized using the red dextran appearing between the cells. Scale bar = 10 μm.
